# Training in regional anaesthesia: there is much to do

**DOI:** 10.1016/j.bjao.2024.100260

**Published:** 2024-01-30

**Authors:** Emmanuel Boselli

**Affiliations:** 1 Pierre Oudot Hospital Center, Bourgoin-Jallieu, France; 2 University Lyon I Claude Bernard, Lyon, France

**Keywords:** central neuraxial procedures, peripheral nerve block, regional anaesthesia, survey study, teaching, training

## Abstract

This editorial discusses a survey of anaesthesia trainees and trainers from the UK concerning training in regional anaesthesia. The study found a large disparity in the number and diversity of regional anaesthesia procedures carried out by trainees during their initial training and that the presence of a departmental regional anaesthesia training lead improves the quality of teaching of these techniques. This study emphasises the fact that there is still a huge effort required to provide adequate training in regional anaesthetic techniques in the UK if patients are to benefit from the optimal postoperative analgesia they provide: the same probably applies also in many other European countries.

Pain remains common after surgery and regional anaesthesia techniques, including neuraxial and peripheral nerve blocks, combined with multimodal analgesia have demonstrated their efficacy to decrease immediate postoperative pain scores.^1^ The survey by Bellew and colleagues^2^ published in the December 2023 issue of *BJA Open* adds to the literature supporting the notion that clinical experience and access to training in regional anaesthesia are of paramount importance to ensure optimal postoperative analgesia for patients. It also underlines that having departmental regional anaesthesia lead clinicians improves equity and quality in training opportunities.

This study evaluated survey responses from 492 trainees (corresponding to a response rate to the survey of 19.2%) and 114 tutors from the UK (corresponding to a response rate of 45.2%). The results show that most trainees were trained in epidural (84%) and spinal (93%) obstetric anaesthesia, but this rate decreased to 65% in non-obstetric spinal and to <10% in non-obstetric epidural anaesthesia. The rate of trainees having performed >20 procedures was also somewhat low for upper limb (31%), lower limb (45%), chest wall (5.3%), and abdominal wall (16%) blocks. However, there was a positive association between having a lead clinician for regional anaesthesia and reported confidence to provide regional anaesthesia training at all stages of the curriculum.

These data expose the current shortfall in training opportunities in regional anaesthesia for anaesthesia trainees in the UK and emphasise the fact that there is still a huge effort required if sufficient training is to be delivered to provide optimal postoperative analgesia for patients using regional anaesthesia. The recent European Society of Anaesthesiology and Intensive Care (ESAIC) guidelines have provided some recommendations for competency assessment in ultrasound-guided regional anaesthesia procedures.^3^ All the ESAIC experts agreed that in order to gain some proficiency before a graduated introduction to learning in the clinical setting, ultrasound training should begin with the acquisition of suitable classroom-based knowledge and practical activities (lectures, video demonstrations, one-to-one sessions, e-learning tools, and simulated practice) and also maybe cadaver workshops. These guidelines also underline the fact that proper training requires a structured training programme, clinical learning opportunities and an appropriate patient and teacher. They state that before attempting their first directly supervised attempt for each ultrasound-guided regional anaesthesia procedure, the trainees should have observed five ultrasound-guided procedures of that type and performed five ultrasound scans on patients scheduled for that ultrasound-guided procedure. Subsequently, for each ultrasound-guided regional anaesthesia procedure, the trainees should be directly observed for at least five procedures of that type before they perform the procedure with more distant supervision.

The current survey by Bellew and colleagues^2^ confirms the importance of clinical experience and access to training in regional anaesthesia and supports the introduction of departmental regional anaesthesia leads to improve equity and quality in training opportunities. It would also be of interest to know how representative the UK experience is of training in regional anaesthesia in other countries. To my knowledge, there is no official training programme in regional anaesthesia in France—and probably in other European countries—apart from providing some theoretical courses, practical training, and workshops for trainees during their residency. A European survey on regional anaesthesia training may help to provide a more precise overview on European practice in this field.

Bellew and colleagues^2^ have made an important observation, but there remains an enormous workload to develop regional anaesthesia, if these analgesia techniques are to become the reference for all appropriate surgical procedures. To provide the required number of training opportunities and expert trainers will require substantial human and financial resources but, perhaps above all, institutional will.

## Declarations of interest

B-Braun and Sonosite have provided needles and ultrasound machines for EB to use for regional anaesthesia teaching workshops.
